# Actigraphy monitoring in anxiety disorders: A mini-review of the literature

**DOI:** 10.3389/fpsyt.2022.984878

**Published:** 2022-08-03

**Authors:** Martin Pastre, Jorge Lopez-Castroman

**Affiliations:** ^1^Department of Psychiatry, CHU Nimes, Nimes, France; ^2^Institut de Génomique Fonctionnelle (IGF), Université de Montpellier, CNRS, INSERM, Montpellier, France; ^3^Centro de Investigacion Biomedical en Salud Mental (CIBERSAM), Instituto de Salud Carlos III (ISCIII), Madrid, Spain

**Keywords:** physical activity, circadian rhythm, sleep disturbances, wearable sensor, polysomnography, phobic and anxiety disorders

## Abstract

Sleep disturbances and changes of activity patterns are not uncommon in anxiety disorders, but they are rarely the object of attention. Actigraphic monitoring of day and night activity patterns could provide useful data to detect symptom worsening, prevent risk periods, and evaluate treatment efficacy in those disorders. Thus, we have conducted a systematic search of the scientific literature to find any original study using actigraphic monitoring to investigate activity and sleep patterns in patients affected by any type of anxiety disorder according to the definition of the DSM-5. We found only six studies fulfilling these criteria. Three studies report significant findings in patients suffering from anxiety disorders. Overall, the samples and methods are heterogeneous. Although the authors support the interest of actigraphic monitoring in anxiety disorders, the evidence to date is very limited.

## Introduction

Anxiety disorders are prevalent and disabling conditions characterized by excessive fear or anxiety, as well as a range of other cognitive and somatic symptoms. Comorbidity with other anxiety disorders and other mental disorders is very frequent, as well as with non-psychiatric medical conditions (1). Large epidemiological samples have estimated their lifetime prevalence at 14.5% in Europe (2) and 33.7% in the US (3), but these numbers comprise obsessive-compulsive and related disorders and trauma and stressor-related disorders, which no longer belong to the category of anxiety disorders in the DSM-5 (4). The 12-month prevalence reported in DSM-5 for adult anxiety disorders ranges from 1 to 3% in the case of panic disorder (PD), 2–7% for social phobia, 0.4–9% for generalized anxiety disorder (GAD), 1–2% for agoraphobia and 6–9% for specific phobias, with the highest prevalence rates being generally reported in the US (4). The wide ranges in these figures are due, among other reasons, to differences in the diagnostic assessment methods and the target populations [for a detailed review see Bandelow and Michaelis (5)]. The 2019 Global Burden of Disease study provides an estimate of the disability associated with these disorders, which are the 24th leading cause in disability-adjusted life-years (the 6th in young people aged 10–24 years) (6).

Among the symptoms of anxiety disorders, sleep disturbances and changes in physical activity (PA) patterns are rarely the object of attention. Contrary to depression, these symptoms are not part of the diagnostic criteria except for GAD, which includes an item about sleeping difficulties, but all anxiety disorders seem to be associated with some degree of sleep disturbances and changes in PA. With regards to sleep, a recent meta-analysis (7) based on polysomnography or self-reported sleep data in controlled studies has shown that patients suffering from anxiety disorders have less sleep continuity (Hedge's *g* = −0.49), an average of 21 min less in total sleep time (*g* = −0.40) and more subjective sleep disturbances (*g* = 2.16) compared to healthy controls with no mental disorder. It should be noted that GAD patients reported the highest scores of subjective sleep disturbances (*g* = 5.55).

Concerning PA patterns, anxiety disorders are characterized by excessive daytime arousal or restlessness according to heart rate and activity monitoring (8). Excessive arousal however does not imply more PA. Two recent meta-analyses found that anxiety disorders and anxiety symptoms are associated with sedentary behavior (9, 10). Also, symptomatic forms of anxiety were prospectively associated with less PA two years later according to a large epidemiological survey with almost 3,000 persons in the Netherlands (11). The association seems to be bidirectional since low sports participation at baseline was associated with symptomatic anxiety two years later. This has implications for treatment. A meta-analysis of randomized controlled trials proved that both aerobic and anaerobic activity reduces the intensity of anxiety symptoms (12), and PA has been proposed as an effective adjunctive treatment for anxiety disorders (13).

Actigraphy can be used to monitor activity rhythms and sleep in mental disorders (14). Actigraphic devices can be routinely used in daily life, have a limited cost compared to polysomnography, and they provide quantifiable objective data that substantially improve the utility of self-reported measures (15). A recent retrospective study investigated the phenomenon of “misperception of sleep” (discrepancies between objective and subjective measures of sleep), and showed that it is a common feature in anxiety disorders (16). Considering all the above and the absence of any review on the topic, we decided to conduct a systematic review of scientific papers using actigraphic monitoring to measure activity patterns and sleep in anxiety disorders.

## Methods

### Selection of studies

We selected all studies according to the following eligibility criteria: (i) original studies published until June 2022 in English, French or Spanish language, (ii) actigraphy measures were used for activity monitoring, including activity patterns during the day and/or sleep parameters at night, (iii) the study samples comprised adult patients with any anxiety disorder diagnosis included in the corresponding DSM-5 category. Therefore, studies investigating trauma or stress-related disorders and obsessive-compulsive and related disorders were excluded. We also excluded studies in which the diagnostic criteria of anxiety disorders were not clearly respected (for example, when only anxiety symptoms were reported) or the actigraphic monitoring was limited to an experimental procedure in a clinical setting (not reflecting daily activity). We followed PRISMA 2020 checklist for systematic reviews and published the protocol on the PROSPERO registry for systematic reviews (CRD42022323708).

### Data sources and search strategy

To identify potential papers, we searched three databases: PubMed, WebOfScience, and PsycINFO until June 2022 with the following equation terms: [(“Anxiety Disorders” OR “Social Anxiety” OR “Generalized Anxiety Disorder” OR “Panic disorder” OR “Social, Phobia” OR “phobic disorder” OR “Phobia, Specific” OR agoraphobia) NOT (“Obsessive-Compulsive Disorder” OR “Anxiety, Separation” OR “Neurocirculatory Asthenia” OR “Neurotic Disorders”)] AND (actigrap^*^ OR actimet^*^ OR actograp^*^ OR actomet^*^ OR accelerometer).

The title and abstract of each potential paper were screened by two reviewers working independently (MP and JLC). Zotero software was used for the management of records. The full text of eligible studies was then reviewed independently by the same two reviewers to assess all inclusion and exclusion criteria.

### Data extraction and quality assessment

We extracted all relevant data from selected papers using a data chart. The quality of each study was assessed using a modified version of the Effective Public Health Practice Project Tool (17) that we built for the purpose of this review. We used the EPHPP tool because the designs of selected studies were highly heterogeneous. Sections D, G, and H of the original scale were not relevant because our review did not include any interventional study and were therefore suppressed from the global rating. Likewise, section C was also revised because the first part of the section (Q1, “Were there important differences between groups prior to the intervention?”) was not applicable. Thus, we considered only the second part of the section (Q2, “Indicate the percentage of relevant confounders that were controlled”) for the rating of section C.

## Results

**Figure 1:**
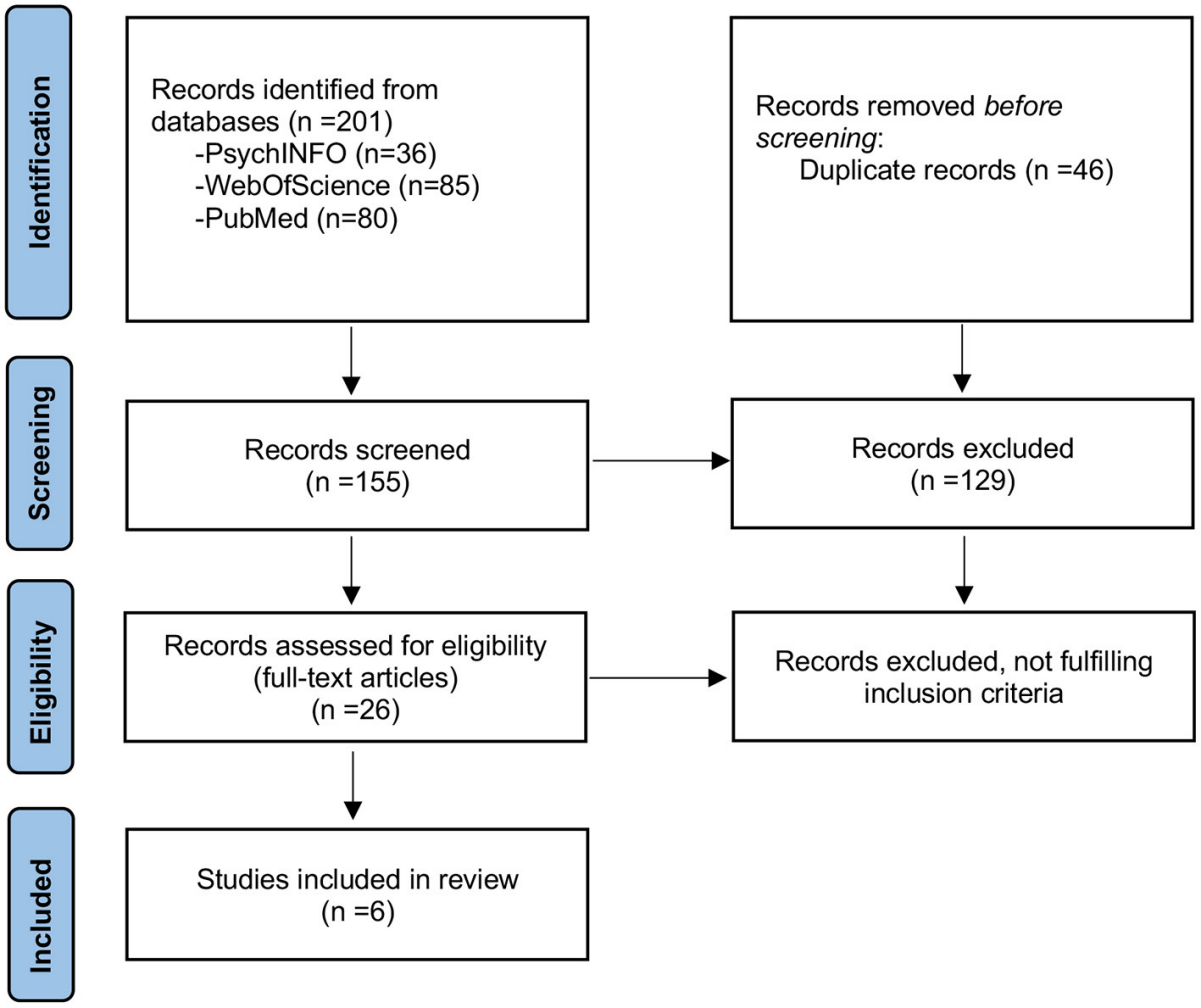
Flowchart of selected studies.

The search retrieved 201 potential papers (80 from PubMed, 85 from WebOfScience, and 36 from Psychinfo). After the removal of duplicates (46 papers), 129 articles were excluded based on title or abstract (not relevant to the topic). Among the 26 papers that were read in full to assess eligibility, 20 were excluded because they did not fulfill the criteria. Most of the excluded papers focused on anxiety symptoms only and did not consider anxiety disorder diagnoses. Three papers (15, 18, 19) used pooled diagnostic data of anxiety disorders and mood disorders. We contacted the authors to obtain specific data on anxiety disorders, but we did not receive an answer. Six articles were included in the mini-review (see Table 1 for a summary of principal results). A flow diagram based on PRISMA 2020 guidelines is shown in Figure 1. Overall, the studies presented a substantial risk of bias (see Supplementary Table 1 for quality assessment).

**Table 1:** Description of selected studies.

	**Sample**	**Comparison group**	**Type of anxiety disorder**	**Outcomes**	**Main results**
Helgadóttir et al. (25)	22	Patients with major depression or comorbid anxiety and depression	GAD, PD, SAD, (PTSD)	Average counts per min, % of sedentary bouts, % of activity bouts, total time in sedentary bouts, number of sedentary bouts	No significative differences in physical activity patterns between depressive and anxious participants
Koolhaas et al. (24)	147 + 59^*^	Populational cohort	GAD, PD, AgPh, SAD, SPh	Hours per day of sedentary behavior (defined as <199 count per min during waking hours)	Cross-sectionally: no significative association between anxiety disorders and sedentary behavior after adjustment on cofounders. Longitudinally: no significative association between sedentary time and subsequent development of anxiety disorder
Luik et al. (20)	144	Populational cohort	GAD, PD, AgPh, SAD, SPh	Fragmentation of the rhythm, stability of the rhythm over days, timing of the rhythm. TST, sleep onset latency and WASO	Anxiety disorders associated with more fragmented rhythm (intradaily variability), independent of covariates (OR: 1.39 per 1 SD, 95% CI: [1.13; 1.70], *p =* 0.002). GAD (n = 39) associated with more fragmented rhythms (OR: 1.75 per 1 SD, 95% CI: [1.20; 2.55], *p =* 0.004), but also shorter TST (OR: 0.66 per 1 h, 95% CI: [0.45; 0.97], *P* = 0.033)
Sakamoto et al. (27)	16	None	PD	Mesor, circadian amplitude, acrophase	Association between frequency of panic attacks and mesor (*r =* 0.55, *p =* 0.03), and between HAM-A score and mesor (*r =* 0.62, *p =* 0.01)
Todder and Baune (21)	15	Healthy controls	PD	Sleep time (%), sleep efficiency (%), index of fragmentation of sleep	No significative difference between patients and controls, or before and after treatment by escitalopram
Wainberg et al. (22)	4847	Psychiatric outpatients	GAD, PD, AgPh, SAD, SPh	Sleep efficiency, longest sleep bout, wake-up/bed-time, WASO, number of awekenings, number of naps, bedtime variability, sleep duration variability	Anxiety disorders associated with sleep disturbances: WASO (beta coefficient for linear regressio*n =* 0.04), later bed-time (0.03), later wake-up time (0.04), sleep efficiency (−0.05), number of awakenings (0.04), longest sleep bout (−0.04), number of naps (0.04), bedtime variability (0.03) and sleep duration variability (0.04)

* They were 147 prevalent cases of anxiety disorders used in the cross-sectional analysis, and 59 incident cases used in the longitudinal analysis. PD, Panic Disorder; GAD, Generalized Anxiety Disorder; SAD, Social Anxiety Disorder; SPh, Specific Phobia; AgPh, Agoraphobia; PTSD, Post Traumatic Stress Disorder.

Hereafter we describe the six included studies according to their quality score (highest quality studies are presented first).

Todder and Baune (21) followed prospectively a cohort of 15 women with PD before and after the instauration of the antidepressant escitalopram (up to 10 mg/day), seeking changes in actigraphic parameters of sleep. There was a “wash-out” period followed by 4 weeks of treatment with continuous actigraphic monitoring. Assessment scales such as the Panic and Agoraphobic scale, the Hamilton anxiety scale (HAM-A) and the Pittsburgh sleep quality index were completed once per week. Patients under benzodiazepine treatment were excluded. The Mini-International Neuropsychiatric Interview (MINI) inventory was used to confirm the diagnoses. Night-time activity was characterized by sleep time (percentage of time asleep between onset and end of sleep), and sleep efficiency (ratio between actual sleep time and total time in bed). These outcomes did not change after treatment and did not differ with those of a control group of female healthy administration workers. At 4 weeks, there was no significant difference in sleep patterns between patients that showed a clinical improvement (>50% HAM-A score) and those that did not.

The study by Luik et al. (20) analyzed cross-sectionally the circadian activity and sleep patterns of patients with anxiety disorders in a populational cohort (>45 years old) from the Rotterdam Study (23). 96 h of actigraphic data was collected from 1,714 people. Anxiety disorders (*n* = 141) were diagnosed using the Composite International Diagnostic Interview (CIDI). Nonparametric measures were used to assess activity rhythms, namely: interday stability, intraday variability (indicative of rhythm fragmentation, i.e., transitions from an active to an inactive state), and dominant rest phase onset (start time of lowest activity period). Concerning sleep, total sleep time (TST), sleep onset latency and wake after sleep onset (i.e., time periods of wakefulness after sleep onset, WASO) were recorded. The reference category for logistic regression analysis comprised participants with no clinical symptoms of depression or anxiety (*n* = 1,441). There was a significant association between fragmented rhythms and the prevalence of anxiety disorders, independently of covariates (OR: 1.39 per 1 SD of intradaily variability, [1.13; 1.70], *p* = 0.002). The significant difference persisted after the exclusion of 47 patients with anxiety disorders and substantial depressive symptoms (Center for Epidemiologic Studies-Depression scale >15). The authors also found that GAD (*n* = 39) was associated with more fragmented rhythms (OR: 1.75 per 1 SD, [1.20; 2.55], *p* = 0.004) and a shorter TST (OR: 0.66 per 1 h, [0.45; 0.97], *p* = 0.033) than the reference group after adjusting on covariates.

Koolhaas et al. (24) studied the relationship of anxiety disorders and sedentary behavior with the data of the Rotterdam Study. A subsample of participants was monitored with an actigraph for a period of 7 days (*n* = 1,841). Activity level during waking hours was measured by the number of counts per minute (a count corresponding to a single movement in any direction captured by the actigraphic sensors). For a given subject, the time of sedentary behavior corresponds to the time during which the activity is <199 counts per min. Diagnoses of anxiety disorders were obtained at baseline using the CIDI. Participants with anxiety disorders (*n* = 147 prevalent cases) reported significantly more sedentary time than the rest of the sample in unadjusted analyses, but after controlling for lifestyle factors (namely disability, smoking, and occupational status) the association did no longer exist. Of note, sedentary behavior at baseline was not associated with the emergence of anxiety disorders during the average 5.7 years of follow-up time (*n* = 59 incident cases).

Wainberg et al. (22) conducted a *post-hoc* cross-sectional analysis of 89 000 individual actigraphic data from the UK-Biobank (a community-based prospective cohort study) to study sleep parameters in anxiety disorders. Anxiety disorders were identified through registered codes of the International Classification of Diseases-10th edition (F40, F41). Several sleep features were measured: bed and wake-up times, sleep duration (defined here as the total duration of night sleep bouts), WASO, sleep efficiency, number of awakenings, duration of longest sleep bout, number of naps, and variability in bedtime/in sleep duration. The presence of any anxiety disorder was associated with sleep disturbances, but effect sizes were small. Compared to healthy participants, patients with anxiety disorders presented a longer WASO (with a beta coefficient for linear regression of 0.04), as well as longer bedtime and wake-up time (0.03 and 0.04 respectively). The same pattern was observed for bedtime variability and sleep duration variability. Sleep efficiency (−0.05) and the duration of the longest sleep bout (−0.04) was decreased, and they experienced more awakenings (0.04).

Helgadóttir et al. (25) used actigraphic data of 165 anxious and/or depressed Swedish adults from the Regassa randomized controlled study (26) to investigate their level of sedentary behavior. All participants, who had a minimum score of 10 on the Patient Health Questionnaire, wore an actigraph for seven days. Diagnoses were obtained with the MINI. They measured activity level using the same proxy described above (number of counts per minute), considering <100 counts per min during twenty consecutive minutes as a sedentary activity bout, 100–1,951 counts per minute as light PA, and more than 1,951 counts per min as moderate to vigorous activity. They then calculated the total time spent in sedentary bouts, as well as the number of sedentary bouts. Twenty-two participants were diagnosed with an anxiety disorder, while 121 had depressive and anxiety disorders at the same time. All the participants were rather sedentary, but there was no statistical difference in activity measures between diagnostic groups.

Finally, Sakamoto et al. (27) investigated the effect of PD severity on 24h activity patterns in 16 outpatients. The participants were recruited through advertisements and assessed with the HAM-A and the Panic Disorder Severity Scale on the first day of the study. They all received a diagnosis of PD with agoraphobia (DSM-IV). Only two patients were male. Most of them were treated with antidepressants and/or benzodiazepines. The patients used electronic diaries (watch-type computers) for 14 days to note information on any panic attack. Also, the intensity of the symptomatology was actively assessed with daily ecological momentary assessment questions. Investigators ran a “cosinor” analysis to describe the timing and amplitude of PA, considered as a circadian process with a particular rhythm. This model provides the estimation of the “mesor” (or corrected amplitude mean) of the circadian rhythm as well as the “acrophase” (peak time in the model). Pearson's correlation analysis showed a significant association between the mesor (from double cosinor analysis) and the frequency of panic attacks (*r* = 0.55, *p* = 0.03) as well as the mesor and the HAM-A score (*r* = 0.62, *p* = 0.01).

## Discussion

A large share of the recent literature about actigraphic measures in psychiatry is focused on mood disorders. In contrast, we decided to review systematically the objective alterations of sleep and activity patterns associated with anxiety disorders that so far have been the object of only a handful of studies. Although the results of our review show that these symptoms can be objectively detected in anxiety disorders, for the moment there is very limited evidence supporting the use of actigraphic measures to monitor their evolution or severity.

Four papers studied anxiety disorders as a general category. All of them were secondary studies, based on large datasets. Luik et al. (20) found fragmented 24 h circadian rhythm measures in anxiety disorders and specifically in GAD, which was also associated with shorter TST. Participants diagnosed with anxiety disorders in the UK-Biobank were more likely to have a disturbed sleep (i.e., notably higher WASO, more awakenings and less sleep efficiency) than healthy controls, but this pattern was shared across psychiatric conditions. These results are consistent with previous self-reported or polysomnographic data regarding altered sleep continuity and lower TST in anxiety disorders (7). In contrast, Koolhaas et al. (24) did not find any association, either cross-sectionally or longitudinally, between diurnal sedentary behavior and anxiety disorders, contradicting studies based on self-reports (9, 10). According to the authors, this discrepancy can be explained by the insufficient precision of actigraphic measures and the fact that previous studies did not control for important confounders, such as disability or occupational status. Helgadóttir et al. (25) also failed to find any differences in sedentary behavior when comparing anxiety-disordered participants and those suffering from depression or comorbid anxiety and depression.

The physiopathological relationship between sleep disturbances and anxiety can be better understood with the results of a recent study. The anxiety symptoms that emerged in patients submitted to sleep deprivation were associated in functional MRI with an hypoactivity of the medial prefrontal cortex, involved in emotional control, and an hyperactivity in the amygdala and dorsal anterior cingulate cortex, responsible of the reactivity to negative emotions (28). Also, the amount of slow-wave sleep predicted the reactivation of medial prefrontal cortex the next day, suggesting an anxiolytic effect of this particular phase of sleep that is known to be shortened in patients suffering from GAD (29) and PD (30).

Some actigraphic studies focused on specific anxiety disorders. Todder and Baune (21) expected to find an actigraphic marker of the clinical response to antidepressants in PD but they did not find any association with sleep disturbances. Sakamoto et al. (27) showed, by using a proxy to measure circadian amplitude in a clinical sample of panic disorders with phobic avoidance, that patients with a more severe form of panic disorder showed greater motor activity. In the same way, a study using a motion sensor found that PD patients with a higher level of phobic avoidance had greater motor activity than controls and PD patients with a lower level of avoidance (8). In the case of GAD, a recent paper (31) investigated restlessness in patients with this diagnosis and healthy controls using actigraphy through a threat-exposure task. Restlessness, which is a subjective feeling close to hyperarousal, is a core feature of GAD and one of its diagnostic criteria. In this study the GAD group did not show greater actigraphic movement magnitude than controls, despite having a significantly higher self-reported restlessness level at baseline and during the threat exposure. Participants with restlessness had a significantly heightened movement level at baseline and through the stages of the increasingly threatening task compared to those without. Moreover, objective measures failed to confirm the subjective restlessness reported by people with GAD, a contradiction that was described by the authors as the “reactivity paradox”: self-reported restlessness does not match objective measures of threat reactivity (31). Overall, these findings suggest that restlessness in GAD could constitute a chronic state of arousal rather than a tendency to overreact while anticipating or being exposed to a threat.

In this review, we excluded papers based on patients presenting anxiety symptoms only because of the transdiagnostic and unspecific nature of these symptoms. However, anxiety symptoms can also impair sleep and activity features. Spira et al. (32) in a sample of older adults with primary insomnia, showed that trait anxiety was associated with greater actigraphy-measured WASO. Studies with pooled samples of depressive and anxiety disorders were also excluded, although we retrieve in clinical samples with this comorbidity the same types of activity and sleep alterations patterns as for anxiety disorders alone. By monitoring sleep, circadian rhythm and PA in a sample of 359 participants with anxiety and/or depressive disorders, Difransesco et al. (15) found that currently anxious and/or depressed patients were less active (with a lower circadian relative amplitude between day-time and night-time activity levels) than controls. Interestingly, the more severe the symptoms of anxiety and depression, the lower the level of PA and the relative amplitude of circadian rhythms. In the same study, participants diagnosed with anxiety and/or depressive disorders reported more insomnia and longer sleep duration, but this difference was not present with objective measures.

Actigraphy has also been used as a prognostic biomarker in the field of anxiety disorders. Jacobson et al. (33) investigated actigraphic measures of movement patterns in GAD and PD. Participants were followed up to 18 years, and a deep learning model based on various activity and sleep features could predict symptom worsening over time with an AUC = 0.696 (84.6% sensitivity, 52.7% specificity). The same authors found with passive data from a wearable accelerometer that patients with higher social anxiety symptoms had lower movement amplitudes (34).

There are several limits to the scope of this review. First, each anxiety disorder might have distinct sleep and activity patterns, despite their common physiopathology and very frequent comorbidity, but most of the included studies considered them in a single category. Second, the restricted number and quality of the studies precludes any strong interpretation of the existing evidence. We were unable to obtain detailed data on the studies that pooled depressive and anxiety disorders. Furthermore, only two of the selected studies (20, 21) took into account the potential interaction of benzodiazepine and/or antidepressant medication in the relationship between sleep and anxiety, despite their wide prescription in anxiety disorders (and particularly among anxious patients presenting sleep disorders) and the well-documented effects of these drugs on sleep architecture (35, 36).

In summary, few studies have yet examined objectively sleep and daily activity changes associated with anxiety disorders. The extant studies are heterogeneous, with an overall high risk of bias. Only half of those included report statistically significant results linking anxiety disorders with disturbances in sleep and activity patterns, and the results are sometimes conflicting. Overall, we want to point out the need for new and specific research in the field, given the burden caused by these disorders and the potential interest of ecological interventions, i.e., based on daily life activities, to improve their prognosis. Characterizing activity and sleep change patterns in people suffering from anxiety disorders might provide useful knowledge to monitor the effects of pharmacological and behavioral interventions.

## Author contributions

MP and JL-C reviewed the articles and drafted the manuscript. All authors contributed to the conception and design of the study and approved the final version.

## Conflict of interest

The authors declare that the research was conducted in the absence of any commercial or financial relationships that could be construed as a potential conflict of interest.

## Publisher's note

All claims expressed in this article are solely those of the authors and do not necessarily represent those of their affiliated organizations, or those of the publisher, the editors and the reviewers. Any product that may be evaluated in this article, or claim that may be made by its manufacturer, is not guaranteed or endorsed by the publisher.

## Abbreviations

GAD: Generalized Anxiety Disorder
PA: Physical Activity
PD: Panic Disorder
TST: Total Sleep Time
WASO: Wake After Sleep Onset.
