# Supplementary material for: Actigraphy monitoring in anxiety disorders: A mini-review of the literature
Source: Front Psychiatry. 2022 Aug 3;13:984878. doi: 10.3389/fpsyt.2022.984878 (PMC9381974; doi:10.3389/fpsyt.2022.984878)
Supplement: Supplementary file 1 [file Table_1.DOCX]

**S1Table 1:** Quality assessment of selected studies using a modified version of EPHPP Quality Assessment Tool for Quantitative Studies (18).

| EPHPP Quality Assessment Tool  for Quantitative Studies | Journal | Number of subjects | Selection | Study design | Cofounders | Data collection methods | Drop-outs | Global quality |
| --- | --- | --- | --- | --- | --- | --- | --- | --- |
| Helgadottir et al., 2019 | PLoS Medicine | 22 | 3(3,5) | 3 (7 : cross-sectional) | 2 | 1(1,1) | NA (4,5) | 3 |
| Kolhaas et al., 2019 | Mental Health and Physical Activity | 147+59* | 3(3,2) | 2/3 (7 : cross-sectional + 3)  (mixed design) | 1 | 1(1,1) | 3(2,4) | 3 |
| Luik et al., 2015 | Depression and Anxiety | 144 | 2(2,2) | 3 (7 : cross-sectional) | 1 | 1(1,1) | NA (4,5) | 2 |
| Sakamoto et al., 2018 | BioPsychoSocial Medicine | 16 | 3(3,5) | 3 (7 : cross-sectional) | 3 | 1(1,1) | NA (4,5) | 3 |
| Todder & Baune, 2010 | Human Psychopharmacology | 15 | 2(2,5) | 2 (3) | 2 | 1(1,1) | 1(1,1) | 1 |
| Wainberg et al., 2021 | PLoS Medicine | 4847 | 3(4,5) | 3 (7 : cross-sectional) | 2 | 1(1,1) | NA (4,5) | 3 |

Global ratings range from 1 to 3 (interpretation of the quality: 1 good, 2 fair and 3 poor). The ratings are determined by the scores from the different sections (same interpretation for each section). Detailed scores to each question are shown in parenthesis (see EPHPP rating tool and manual for detailed question items). NA= not applicable. *They were 147 prevalent cases of anxiety disorders used in the cross-sectional analysis, and 59 incident cases used in the longitudinal analysis.
